# Impact of a delayed second dose of mRNA vaccine (BNT162b2) and inactivated SARS-CoV-2 vaccine (CoronaVac) on risks of all-cause mortality, emergency department visit, and unscheduled hospitalization

**DOI:** 10.1186/s12916-022-02321-4

**Published:** 2022-03-17

**Authors:** Carlos King Ho Wong, Xi Xiong, Kristy Tsz Kwan Lau, Celine Sze Ling Chui, Francisco Tsz Tsun Lai, Xue Li, Esther Wai Yin Chan, Eric Yuk Fai Wan, Ivan Chi Ho Au, Benjamin John Cowling, Cheuk Kwong Lee, Ian Chi Kei Wong

**Affiliations:** 1 Centre for Safe Medication Practice and Research, Department of Pharmacology and Pharmacy, LKS Faculty of Medicine, The University of Hong Kong, Pok Fu Lam, Hong Kong SAR, China; 2 Department of Family Medicine and Primary Care, School of Clinical Medicine, LKS Faculty of Medicine, The University of Hong Kong, Hong Kong SAR, China; 3 Laboratory of Data Discovery for Health (D24H), Hong Kong Science and Technology Park, Sha Tin, Hong Kong SAR, China; 4 School of Nursing, Li Ka Shing Faculty of Medicine, The University of Hong Kong, Hong Kong SAR, China; 5 School of Public Health, Li Ka Shing Faculty of Medicine, The University of Hong Kong, Hong Kong SAR, China; 6 Department of Medicine, School of Clinical Medicine, Li Ka Shing Faculty of Medicine, The University of Hong Kong, Hong Kong SAR, China; 7 WHO Collaborating Centre for Infectious Disease Epidemiology and Control, School of Public Health, Li Ka Shing Faculty of Medicine, The University of Hong Kong, Hong Kong SAR, China; 8 Hong Kong Red Cross Blood Transfusion Service, Hospital Authority, Kowloon, Hong Kong SAR, China; 9 Research Department of Practice and Policy, UCL School of Pharmacy, University College London, London, UK

## Abstract

**Background:**

Safety after the second dose of the SARS-CoV-2 vaccine remains to be elucidated, especially among individuals reporting adverse events after their first dose. This study aims to evaluate the impact of a delayed second dose on all-cause mortality and emergency services.

**Methods:**

A territory-wide, retrospective cohort of people who had completed two doses of mRNA (BNT162b2) or inactivated SARS-CoV-2 (CoronaVac) vaccine between February 23 and July 3, 2021, in Hong Kong was analyzed, with linkage to electronic health records retrieved from the Hong Kong Hospital Authority. Vaccine recipients were classified as receiving a second dose within recommended intervals (21–28 days for BNT162b2; 14–28 days for CoronaVac) or delayed. Study outcomes were all-cause mortality, emergency department (ED) visits, and unscheduled hospitalizations within 28 days after the second dose of vaccination.

**Results:**

Among 417,497 BNT162b2 and 354,283 CoronaVac second dose recipients, 3.8% and 28.5% received the second dose beyond the recommended intervals (mean 34.4 and 31.8 days), respectively. During the study period, there were < 5 daily new cases of COVID-19 infections in the community. Delaying the second dose was not associated with all-cause mortality (hazard ratio [HR] = 1.185, 95% CI 0.478–2.937, *P* = 0.714), risk of ED visit (HR = 0.966, 95% CI 0.926–1.008, *P* = 0.113), and risk of unscheduled hospitalization (HR = 0.956, 95% CI 0.878–1.040, *P* = 0.294) compared to that within the recommended interval for CoronaVac recipients. No statistically significant differences in all-cause mortality (HR = 4.438, 95% CI 0.951–20.701, *P* = 0.058), ED visit (HR = 1.037, 95% CI 0.951–1.130, *P* = 0.411), and unscheduled hospitalization (HR = 1.054, 95% CI 0.867–1.281, *P* = 0.597) were identified between people who received a second dose of BNT162b2 within and beyond the recommended intervals.

**Conclusions:**

No significant association between delayed second dose of BNT162b2 or CoronaVac and all-cause mortality, ED visit, and unscheduled hospitalization was observed in the present cohort. Regardless of the recommended or delayed schedule for SARS-CoV-2 vaccination, a second dose of both vaccines should be administered to obtain better protection against infection and serious disease. The second dose should be administered within the recommended interval following the manufacturer’s product information, until further studies support the benefits of delaying vaccination outweighing the risks.

**Supplementary Information:**

The online version contains supplementary material available at 10.1186/s12916-022-02321-4.

## Background

Mass vaccination programs have been recognized as a key strategy to achieve high levels of population immunity to mitigate the impact of the coronavirus disease 2019 (COVID-19) pandemic. Hence, various SARS-CoV-2 vaccine candidates have been developed at an unprecedented speed using different platform technologies [1]. While most types of vaccine require a two-dose regimen for adequate protection, some governments have been exploring the option of delaying the second dose to beyond the recommended interval of 3–4 weeks between doses, as a contingency measure to offer at least partial immunity to a larger population in the midst of limited vaccine supply [2, 3]. In fact, the ChAdOx1 nCoV-19 vaccine (AZD1222) has been observed to demonstrate higher efficacy and antibody titers with longer prime-boost intervals, such as receiving the second dose ≥ 12 weeks compared to < 6 weeks after the first dose [4]. A recent study on this viral vectored vaccine has identified even higher antibody titers with extended dosing intervals of up to 44–45 weeks, along with further boosts of both humoral and cellular immune responses following the administration of a third dose 28–38 weeks after the second [5]. Similarly, preliminary evidence suggested higher neutralizing antibody levels with the third dose of CoronaVac (an inactivated SARS-CoV-2 vaccine), administered 6 months compared to 4 weeks after the second dose, regardless of a 2-week or 4-week interval between the first and second doses [6]. The theoretical benefit of delaying the second dose was obvious, achieving higher vaccine uptake in a short time and decreasing COVID-19 infection rate, hospitalization, and death under circumstances of limited vaccine supply.

Delaying the second dose of SARS-CoV-2 mRNA vaccines has been supported by mathematical models to reduce infection, hospitalization, and mortality due to COVID-19, conditioned on factors including the vaccination rate, the efficacy of the first dose, and the level of pre-existing immunity [7, 8]. At the individual level, people may postpone or even refuse the second dose vaccination for various reasons, for example, feeling unwell at the scheduled time or having experienced adverse reactions following the first dose [9]. Such fear or hesitancy is valid in light of some real-life observations suggesting that those with adverse reactions to the first dose are at an increased risk of reporting them again after the second dose, especially of the same side effects [9, 10]. In general, the safety profile of a vaccine includes “all adverse events that could potentially be caused, triggered, or worsened at any time after vaccination,” which may consist of acute or chronic health conditions, such as allergic or anaphylactic reactions, and diseases diagnosed following vaccination [11, 12].

In addition to potentially discouraging timely and complete vaccination for adequate protection, adverse events following vaccination may prompt emergency department (ED) visits or even hospitalization if they are severe enough, for instance, cardiac and neurologic symptoms have been identified as primary causes of these medical encounters after the administration of SARS-CoV-2 vaccines [13–15]. Nevertheless, studies on the utilization of healthcare services following vaccination remain scarce [12, 16–18], and to the best of our knowledge, no clinical trials have specifically examined such outcomes with varying intervals between doses. Therefore, this retrospective study aims to address the research gaps of describing the use of health care services after SARS-CoV-2 vaccination in a real-life setting, when the second dose is administered on a delayed schedule compared to that within the recommended interval.

## Methods

### Data source

This study used anonymized vaccination records of individuals with information on the brand of vaccine, venue for vaccination, and date of administration as retrieved from the Department of Health, with linkage to electronic medical records from the Hong Kong Hospital Authority. Electronic medical records included demographics, registered death, hospital admission, ED visits, drug dispensing records, diagnoses, procedures, and laboratory tests. The combined database has been used for high-quality research on evaluating the safety of SARS-CoV-2 vaccines [15, 19–27].

The study was approved by the Institutional Review Board of the University of Hong Kong/Hospital Authority Hong Kong West Cluster (UW 21-149 and UW 21-138) and the Department of Health Ethics Committee (LM 21/2021). All data were anonymized, and the project was granted a waiver of participant consent.

### Study design and participants

This was a retrospective territory-wide cohort study investigating the impact of a delayed second dose of SARS-CoV-2 vaccines. Individuals aged 16 years or above were identified if they had completed the BNT162b2 or CoronaVac vaccine series between February 23 and July 3, 2021, in Hong Kong SAR, China. The recommended age cutoffs for individuals eligible for vaccination during the study period were under 16 years for BNT162b2 and 18 years for CoronaVac. Individuals who had received only one vaccine dose or mixed vaccine doses were excluded from the current analysis.

The Hong Kong government planned and implemented the territory-wide COVID-19 vaccination programme, which commenced on February 23, 2021, for CoronaVac vaccine and March 6, 2021, for BNT162b2. There were early and late vaccination priority policies for a prime dose of the SARS-CoV-2 vaccine in Hong Kong. Additional file 1: Table S1 lists the SARS-CoV-2 vaccination rollout schedule from February 23 to July 31, 2021 [28–32]. However, there were no second dose vaccination priority policies in the Hong Kong population. According to local recommendations [33], the two doses of BNT162b2 should be administered at least 21 days apart, while the recommended interval was 28 days apart for CoronaVac. The governmental vaccination booking system would automatically arrange the administration schedule for a second dose at the first dose appointment. Individuals could select to receive either BNT162b2 or CoronaVac at the first dose appointment and were flexible to change the second dose schedule. Yet, they were not allowed to switch between brands of vaccine after receiving the first dose. Individuals could choose to receive a delayed second dose, but a date earlier than the recommended interval was not permitted.

### Exposure and follow-up period

In the present study, the recommended interval was defined as 21–28 days for BNT162b2 [34] and 14–28 days for CoronaVac [35, 36] (denoted as “recommended”), according to evidence based on the data from large-scale clinical trials and WHO recommendations. Individuals who received the second dose > 28 days after the first dose were regarded as delayed second dose (denoted as “delayed”). The index date was defined as the date of the second dose administration of either vaccine. The follow-up for each person commenced from the index date until the date of death, or 28 days after the second dose, or the end of the data cutoff date (July 31, 2021), whichever came first.

### Study outcomes

The study outcomes were all-cause mortality, ED visits, and unscheduled hospitalizations 28 days after the second dose. Unscheduled hospitalization referred to the patients who visited EDs and needed urgent diagnostic workup and in-patient treatment.

### Baseline covariates

Our study included a list of individuals’ covariates and clinical factors, comprising age, sex, living district, the month of the first dose vaccination, venue for vaccination, any ED visits between the two doses, previous COVID-19 diagnosis (defined by PCR-confirmed infection), pre-existing comorbidities (Charlson’s index, myocardial infarction, ischemic stroke, transient ischemic attack, congestive heart failure, cerebrovascular disease, chronic obstructive pulmonary disease, diabetes without chronic complication, diabetes with chronic complication, chronic renal failure, ulcers, rheumatoid arthritis and other inflammatory polyarthropathies, malignancy, and metastatic solid tumor), and drug history in the past 90 days (renin-angiotensin-system agents, beta blockers, calcium channel blockers, lipid-lowering agents, antidiabetic drugs, antiplatelets, antidepressants, non-steroidal anti-inflammatory drugs (NSAIDs), drugs for gout, antiepileptic drugs, antiviral drugs, and antibacterial drugs). Disease diagnoses are defined by the International Classification of Diseases, Ninth Revision, Clinical Modification (ICD-9-CM) diagnosis codes listed in Additional file 1: Table S2.

### Statistical analysis

The propensity score was estimated by logistic regression in which the dependent variable was the receipt of a second dose at recommended or delayed dosing interval. Adjusting covariates were the observed personal characteristics and clinical factors aforementioned. The propensity of receiving a second dose on recommended interval versus delayed was used to reduce potential bias due to interval selection. Inverse probability of treatment weights (IPTW) was used to equilibrate the baseline variables of individuals in the recommended interval and delayed groups. The extreme weights (e.g., 1st and 99th percentiles) were truncated to obtain a better balance between the groups [37]. Baseline characteristics were summarized as mean and standard deviation (SD) for continuous variables and percentage for categorical variables. The balance of baseline covariates between the groups was further evaluated using the standardized mean difference (SMD) before and after propensity score weighting, with SMDs < 0.1 implying an optimal balance between the groups.

The total number of events, cumulative incidence rates, and crude incidences per 10,000 person-days after two-dose vaccines were reported in the recommended and delayed groups. IPTW-weighted Cox proportional hazard regression models were fitted to estimate the hazard ratio (HR) and corresponding 95% confidence interval (CI) of outcomes comparing delayed second dose to those receiving it within the recommended interval. Subgroup analysis was conducted by age. In a post-hoc analysis, we repeated analyses to compare the outcomes across recommended, *slightly delayed* (those who received the second dose 29–42 days after BNT162b2 or received the second dose 29–56 days after CoronaVac) and *more delayed* (those who received the second dose > 42 days after BNT162b2 or received the second dose > 56 days after CoronaVac) groups [36, 38, 39]. To account for multiple testing for subgroups, Bonferroni correction was used, so the results were considered statistically significant if *P* < 0.05/24 (number of subgroup analyses) = 0.002 [40]. Sensitivity analyses were conducted to assess the robustness of the results by (i) excluding COVID-19-related outcomes or physical injuries; (ii) including “infinite delayers” into the delayed group and thus comparing the outcomes between the combined delayed group and the recommended group to assess the impact of immortal bias on our findings; as an operational definition, we defined “infinite delayers” as patients who did not receive the second dose and with a follow-up period of at least 3 months; and (iii) using the doubly robust method with inverse-probability-weighted regression-adjustment combination.

All statistical analyses were performed using the STATA version 16.0 (StataCorp LP, College Station, TX). A two-sided significance level of *P* < 0.05 was considered statistically significant.

## Results

**Fig. 1:**
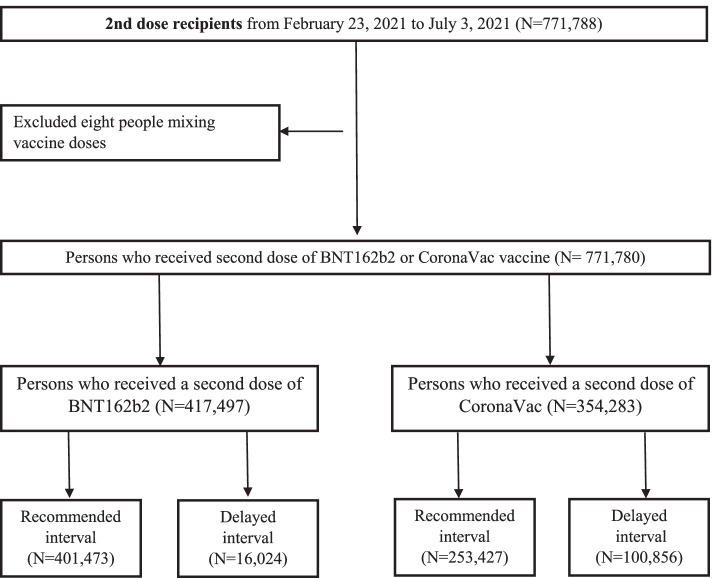
Inclusion and exclusion criteria for analysis of people who received second doses of BNT162b2 or CoronaVac vaccine from February 23, 2021 to July 3, 2021 in Hong Kong SAR, China

Baseline characteristics of fully vaccinated individuals in the two groups by brands of vaccine are presented in Table 1. Among 771,780 individuals administered with two doses of COVID-19 vaccines from February 23 to July 3, 2021, eight receiving BNT162b2 as the first dose followed by CoronaVac were excluded. A total of 654,900 individuals received the second dose within the recommended interval of respective vaccine (401,473 BNT162b2 recipients and 253,427 CoronaVac recipients), while 16,024 (3.8%) and 100,856 (28.5%) individuals received the second dose of BNT162b2 and CoronaVac beyond the recommended intervals, respectively (Fig. 1). There were < 5 daily new COVID-19 infections in the community (500 local cases in 159 days) during the study period, while one new polymerase chain reaction-positive case following the second dose of BNT162b2 vaccination and one COVID-19 case following the second dose of CoronaVac vaccination were reported in our cohort. Among those who received the second dose late, the mean (SD) dosing intervals were 34.4 (8.2) days and 31.8 (6.7) days in the BNT162b2 and CoronaVac groups, respectively (Table 1). Additional file 1: Figure S1 shows the distributions of the second dose interval of BNT162b2 and CoronaVac recipients. The median follow-up was 28 days (interquartile range [IQR] 28–28) for the overall cohort. Additional file 1: Figure S2 shows the distribution of propensity scores by vaccine types. After weighting, all SMDs of baseline characteristics were less than 0.1 (Additional file 1: Table S3).

**Table 1:** Baseline characteristics of people receiving the second dose in recommended or delayed dosing interval by the brand of vaccine before the propensity score weighting

Baseline characteristics	BNT162b2		CoronoVac	
	Recommended (*N* = 401,473)	Delayed (*N* = 16,024)	Recommended (*N* = 253,427)	Delayed (*N* = 100,856)
	Mean ± SD/%	Mean ± SD/%	Mean ± SD/%	Mean ± SD/%
Age, years	47.5 ± 14.9	43.6 ± 14.4	54.8 ± 13.9	53.5 ± 14.2
16–44	44.5%	56.1%	23.2%	26.5%
45–64	41.4%	34.9%	50.7%	50.1%
≥ 65	14.1%	9.0%	26.2%	23.5%
Sex				
Male	48.8%	47.2%	53.0%	50.7%
Female	51.2%	52.8%	47.0%	49.3%
Region				
Hong Kong Island	21.4%	20.5%	13.3%	13.6%
Kowloon	27.1%	27.1%	31.1%	31.6%
New Territories	51.3%	52.2%	55.4%	54.6%
Unknown	0.2%	0.2%	0.2%	0.2%
Dosing interval, days	22.1 ± 2.0	34.4 ± 8.2	28.0 ± 0.0	31.8 ± 6.7
Vaccination site				
Community vaccination centers	99.5%	99.7%	67.3%	50.7%
Clinics	0.0%	0.0%	31.6%	46.7%
Others	0.5%	0.3%	1.0%	2.6%
ED visit between two doses	2.1%	10.0%	2.5%	4.2%
Unscheduled hospitalization between two doses	0.4%	3.4%	0.5%	1.3%
COVID-19 survivor	0.1%	0.1%	0.1%	0.1%
Pre-existing comorbidities				
Charlson’s Index	1.4 ± 1.4	1.1 ± 1.3	2.1 ± 1.5	2.0 ± 1.5
0	35.0%	46.3%	15.9%	18.8%
1–2	41.1%	38.0%	43.9%	45.2%
≥ 3	23.9%	15.8%	40.2%	36.0%
Myocardial infarction	0.1%	0.1%	0.2%	0.1%
Ischemic stroke	0.2%	0.2%	0.4%	0.4%
Transient ischemic attack	0.1%	0.1%	0.2%	0.2%
Congestive heart failure	0.1%	0.0%	0.2%	0.1%
Cerebrovascular disease	0.7%	0.6%	1.4%	1.2%
Chronic obstructive pulmonary disease	0.8%	0.8%	1.0%	1.0%
Diabetes without chronic complication	4.8%	2.9%	7.7%	6.6%
Diabetes with chronic complication	0.1%	0.1%	0.2%	0.2%
Chronic renal failure	0.2%	0.1%	0.4%	0.3%
Ulcers	0.3%	0.2%	0.6%	0.5%
Rheumatoid arthritis and other inflammatory polyarthropathies	0.1%	0.1%	0.1%	0.1%
Malignancy	0.8%	0.6%	0.9%	0.9%
Metastatic solid tumor	0.1%	0.1%	0.1%	0.1%
Drug history				
Renin-angiotensin-system agents	7.0%	4.8%	10.5%	9.5%
Beta blockers	3.9%	2.9%	6.0%	5.2%
Calcium channel blockers	10.4%	6.8%	16.3%	14.5%
Lipid-lowering agents	10.5%	7.6%	16.0%	14.2%
Antidiabetic drugs	4.9%	3.1%	7.9%	6.8%
Antiplatelets	3.1%	2.4%	5.0%	4.5%
Antidepressants	3.0%	2.7%	3.2%	3.2%
NSAIDs	6.3%	7.1%	6.2%	6.5%
Drugs for gout	1.1%	0.8%	1.6%	1.5%
Antiepileptic drugs	1.2%	1.3%	1.1%	1.1%
Antiviral drugs	1.0%	0.9%	1.2%	1.1%
Antibacterial drugs	2.9%	3.7%	2.8%	2.9%

Received second dose 21–28 days (BNT162b2) or 14–28 days (CoronaVac) after the first dose was considered within recommended interval; received second dose 28 days after the first dose was regarded as delayed second dose

*ED* Emergency department, *SD* standard deviation, *NSAIDs* Non-steroidal anti-inflammatory drugs

There were 25,421 individuals (3.3%) who visited EDs after the second dose during the follow-up period (14,453 [3.5%] BNT162b2 recipients, of which 635 [4.0%] had a delayed second dose; 10,968 [3.1%] CoronaVac recipients, of which 3112 [3.1%] had a delayed second dose). The median time from receiving the second dose to ED visits was 10 days (IQR 3–19) for all BNT162b2 recipients, while the median duration was 12 days (IQR for the recommended interval group 5–20; IQR for the delayed group 6–20) in CoronaVac recipients (Table 2). Delaying the second dose was not associated with increased or decreased risk of ED visit compared to individuals receiving it within the recommended interval (BNT162b2: 14.49 vs 12.55 per 10,000 person-days, HR = 1.037, 95% CI 0.951–1.130, *P* = 0.411; CoronaVac: 11.21 vs 11.26 per 10,000 person-days, HR = 0.966, 95% CI 0.926–1.008, *P* = 0.113).

**Table 2:** Number of cases, incidence rates of all-cause mortality, emergency department visits, and unscheduled hospitalizations after second dose of BNT162b2 and CoronaVac

Event	Before weighting										After weighting		
	Recommended (*N* = 654,900)					Delayed (*N* = 116,880)					Delayed vs recommended interval		
	Cumulative incidence		Crude incidence rate (events/10,000 person-days)			Cumulative incidence		Crude incidence rate (events/10,000 person-days)			HR^a^	95% CI	*P*-value
	Cases with event	Rate	Estimate	95% CI	Person-days	Cases with event	Rate	Estimate	95% CI	Person-days			
**Mortality after second dose**													
**BNT162b2**	15	0.0037%	0.0133	(0.01, 0.02)	11,241,070	2	0.0125%	0.0446	(0.01, 0.16)	448,656	4.438	(0.951, 20.701)	0.058
** CoronaVac**	16	0.0063%	0.0225	(0.01, 0.04)	7,095,767	7	0.0069%	0.0248	(0.01, 0.05)	2,823,889	1.185	(0.478, 2.937)	0.714
**ED visit after second dose**													
**BNT162b2**	13,818	3.44%	12.55	(12.34, 12.76)	11,011,002	635	3.96%	14.49	(13.38, 15.66)	438,314	1.037	(0.951, 1.130)	0.411
**CoronaVac**	7856	3.10%	11.26	(11.01, 11.51)	6,977,752	3112	3.09%	11.21	(10.82, 11.61)	2,776,922	0.966	(0.926, 1.008)	0.113
**Unscheduled hospitalization after second dose**													
**BNT162b2**	2929	0.73%	2.62	(2.52, 2.71)	11,195,142	124	0.77%	2.78	(2.31, 3.31)	446,704	1.054	(0.867, 1.281)	0.597
**CoronaVac**	2068	0.82%	2.93	(2.80, 3.06)	7,065,542	781	0.77%	2.78	(2.59, 2.98)	2,812,393	0.956	(0.878, 1.040)	0.294

*HR* Hazard ratio, *CI* Confidence interval, *ED* Emergency department

^a^HR > 1 (or < 1) indicates persons delaying the second dose had a higher risk (or lower risk) of outcome events after the second dose, compared to persons receiving the second dose within the recommended interval

Regarding other outcomes of interest, there were 40 deaths and 5902 unscheduled hospital admissions within 28 days risk period after vaccination. The median time from the second dose administration to unscheduled hospitalization was 11 and 13 days for all BNT162b2 and CoronaVac recipients, respectively. No statistically significant differences in all-cause mortality and risk of unscheduled hospitalization (BNT162b2: 2.78 vs 2.62 per 10,000 person-days, HR = 1.054, 95% CI 0.867–1.281, *P* = 0.597; CoronaVac: 2.78 vs 2.93 per 10,000 person-days, HR = 0.956, 95% CI 0.878–1.040, *P* = 0.294) were identified for people who delayed the second dose of either vaccine. Primary causes of unscheduled hospitalization of people receiving the second dose in recommended or delayed dosing interval by the type of vaccine are shown in Additional file 1: Table S4. There were no marked differences in the top rank of disease diagnosis between the recommended and delayed groups. Unscheduled hospitalization due to “symptoms, signs, and ill-defined conditions” accounted for the most in the recommended and delayed groups for both BNT162b2 and CoronaVac recipients. Four young male (age range 19–35 years) myocarditis cases occurred at 5–20 days after the second dose of BNT162b2 in the recommended group, and no myocarditis cases occurred after the second dose of CoronaVac and delayed second dose of BNT162b2.

Subgroup analyses (Additional file 1: Table S5) showed delaying the second dose had no significant impact on the outcomes for elderly people, consistent with the main analysis. Slightly delaying or more delaying the second dose was not associated with increased or decreased risk of ED visit or unscheduled hospitalization (Additional file 1: Table S6).

The results of sensitivity analyses that excluded COVID-19-related outcomes or physical injuries (Additional file 1: Table S7), included infinite delayers to the delayed group (Additional file 1: Table S8), and used the doubly robust method (Additional file 1: Table S9) were consistent with the result of the main analysis.

## Discussion

This current study examined the safety following the second dose of two different vaccine platforms (BNT162b2, an mRNA vaccine, and CoronaVac, an inactivated virus vaccine), from the aspect of mortality and emergency service utilization, in relation to all adverse events occurring after vaccination that would have been severe or persistent enough to prompt emergency care services. Accordingly, delaying the second dose of BNT162b2 or CoronaVac was not associated with any change in all-cause mortality, risks of ED visit, and unscheduled hospitalization.

While a limited number of studies have explored the alternative dosing regimens with extended prime-boost intervals on the efficacy and immunogenicity outcomes of SARS-CoV-2 vaccines [4, 41–44], comparing delayed to recommended dosing intervals on vaccine safety and utilization of healthcare services has not been addressed in the current literature. In fact, the efficacy and immune responses induced by SARS-CoV-2 vaccines can vary substantially depending on the different platform technologies [1], which would have contributed to the discrepancies in their safety profile. For instance, a systematic review and meta-analysis of clinical trials have found that SARS-CoV-2 mRNA vaccines were associated with relatively higher incidences and risks of adverse reactions versus control, in comparison with vectored or inactivated vaccines, and in particular, for systemic adverse reactions, an increased risk was only observed with mRNA vaccines but not inactivated ones [45]. Therefore, our results should be considered based on the respective vaccine strategies.

As vaccine reactogenicity is likely immune-mediated, it seems logical to expect more side effects of potentially higher severity among individuals with more robust immune responses and inflammation to vaccination [11]. For example, a number of studies have generally concluded that essentially more adverse events, especially systemic reactions, were identified after the second dose of BNT162b2 compared to the first dose, which could be related to the greater immunogenicity following the priming or sensitization to viral antigen with the first dose, and similar associations could be detected with younger age, female sex, individuals with allergies, and COVID-19 survivors [10, 17, 34, 46–53]. Studies had quantified that the amount and lifespan of neutralizing antibodies fell remarkably before the day of the recommended second dose. In contrast, the antibody levels were significantly higher following the second dose of BNT162b2 when the dosing interval was extended to 6–14 weeks instead of the recommended 3–4 weeks, whereas T cell responses were moderately sustained [41, 43, 44, 54]. Therefore, it is proposed that the second encounter of the viral antigens would enhance the immune response when the antibody levels were dropped too low. Yet, a further investigation into the immunogenicity outcomes of delaying the second dose is needed to clarify its impact on reactogenicity, if there is any, and the possible mechanisms involved.

Regarding the inactivated vaccine CoronaVac, data on its safety and efficacy with varying prime-boost intervals remain scarce. One study has recognized that while the seropositive rate was comparable among individuals who had administered their second dose 21 days (recommended) or 1–7 months (extended) after the first dose, the latter group was associated with a significantly lower median antibody level post-vaccination, despite no significant differences were detected with varying extended intervals [42]. Besides, with reference to the platform technology of CoronaVac, antigen immunogenicity of inactivated vaccines could be hampered by the inactivation process, and the antibody titers tend to decrease over time, hence necessitating the administration of several booster doses [1]. While CoronaVac has also been demonstrated to elicit T cell responses in vaccinated individuals [55], the activation and durability of such cellular immunity with varying dosing intervals remain to be verified. Overall, it could be speculated that the immunogenicity of CoronaVac with delayed second dose would unlikely trigger substantial reactogenicity, as evidenced by the comparable risks of medical encounters to those associated with the recommended dosing interval in our study, and in line with studies reporting lower or similar incidences of adverse events following the second dose versus the first [9, 56].

While the relationship between immunogenicity and reactogenicity of SARS-CoV-2 vaccines remains to be elucidated, several studies have observed that a minority of vaccinated individuals did experience adverse reactions that prompted them to seek medical care, at either outpatient clinics or ED, but eventually required hospitalization for further management [10, 12, 16–18, 50]. While some have explored such utilization of healthcare services among vaccinated individuals stratified by the number of doses and any previous SARS-CoV-2 infection, none has described them in relation to varying prime-boost intervals. Further research is needed to determine if the magnitude of inflammation induced by adaptive immune responses of SARS-CoV-2 vaccination parallels the incidence and severity of reactogenicity symptoms [11], with reference to the potential factors aforementioned. In combination with data on the utilization of healthcare services by vaccinated individuals, health professionals can be informed about any trends or specific populations at risk of requiring medical attention and further contribute to monitoring the safety of SARS-CoV-2 vaccination.

Considering the potential waning of immunity from the standard two-dose regimens and the emerging variants of concern, many governments are now initiating the administration of a third dose (or booster) as an attempt to maintain immune protection in the population, especially for the elderly and immunocompromised individuals who are more at risk of severe COVID-19 [57, 58]. Both randomized and observational studies have recently demonstrated that the third dose could significantly enhance the neutralizing antibody responses that have been waning over time among individuals fully vaccinated with two doses, which in turn protect them against SARS-CoV-2 infection, severe COVID-19, and related death, and possibly reducing transmission in the community; these have been observed with both BNT162b2 [59–63] and CoronaVac [6, 58, 64, 65], notably in extended dosing intervals and heterologous vaccination. However, the massive rollout of booster doses remains an ethical controversy given the unequal access and distribution of vaccines worldwide, as well as the lack of long-term data on their safety and efficacy, especially in view of the increasing cases of breakthrough infection with the Omicron variant despite vaccination with booster doses [66]. Moreover, as protection against severe COVID-19 and related death relies mainly on the standard two vaccine doses, further studies are needed to evaluate the cost-effectiveness and optimal timing of booster doses, in addition to any unexpected increases in the incidence of adverse reactions or utilization of healthcare services following vaccination [6, 60, 66].

In this real-life setting, mortality, ED visits, and unscheduled hospitalizations of vaccinated individuals were captured, which were likely contributed by adverse reactions following vaccination that were severe or persistent enough for the people to seek urgent medical care. In contrast to surveillance studies that relied on self-reported adverse reactions, our study would have only included those severe enough to require emergency medical care, which could be an essential piece of information in the evaluation of healthcare services and allocation of resources in the midst of the current pandemic.

Our study is unique in presenting the safety of two different SARS-CoV-2 vaccines via their associated incidences of emergency services and all-cause mortality, based on the comparison of delayed versus recommended dosing intervals. Nevertheless, several limitations of this observational study should also be addressed. First, reasons for delaying the second dose were not available for further analysis, such as mild adverse events not captured by hospital records after the first dose. Hence, the impact of extended dosing interval could not be evaluated in association with the safety of second dose vaccination for individuals developing particular adverse events. Second, our results could be confounded by several residual or unmeasured factors, including but not limited to the lack of data on the utilization of private healthcare services and on individuals who had adverse reactions to the first dose and therefore avoided the second dose totally. Nevertheless, the effect of infinite delayers on our findings was minimal as demonstrated by sensitivity analysis (Additional file 1: Table S8). Furthermore, event occurrence (i.e., ED visits and unscheduled hospitalizations) prior to the second dose were well balanced after the weighting (Additional file 1: Table S3), so confounding effects by the event outcome prior to the second dose were negligible. Third, the lack of immunogenicity data for our study has prevented us from making further inferences about vaccine reactogenicity. The impact of delaying the second dose on COVID-19 infection or vaccine effectiveness was not quantified because of low COVID-19 infection rates during the study period. Fourth, given the relatively small number of more serious events such as deaths and unscheduled hospitalizations, our study would not have the power to distinguish very small differences in event rates, although we are able to confirm that any differences in rates were no greater than 27% for deaths for the BNT162b2 vaccine. Lastly, our results may not be generalizable to other settings, such as those with different population demographics, inadequate vaccine supply, or experiencing COVID-19 outbreaks, as they could confound the utilization of healthcare services and implementation of vaccination strategies.

In conclusion, this study has provided empirical data to describe the trends and populations at risk of mortality and utilizing emergency healthcare services following SARS-CoV-2 vaccination. Delaying the second dose of BNT162b2 or CoronaVac was not associated with increases in all-cause mortality, risk of ED visit, and unscheduled hospitalization compared to vaccination within the recommended interval. Regardless of the recommended or delayed schedule for SARS-CoV-2 vaccination, the second dose of both vaccines should be administered to obtain better protection against infection and serious disease. Second dose should be administered within the recommended interval following the manufacturer’s product information, until further studies support the benefits of delaying vaccination outweighing the risks. Future research should explore the relationship between immunogenicity and reactogenicity of SARS-CoV-2 vaccines in various population subgroups and dosing intervals.

## Supplementary Information

**Additional file 1: **Impact of a delayed second dose of mRNA vaccine (BNT162b2) and inactivated SARS-CoV-2 vaccine (CoronaVac) on risks of all-cause mortality, emergency department visit, unscheduled hospitalization. **Table S1.** Vaccination program priority groups rollout schedule in Hong Kong. **Table S2.** Disease diagnosis defined by International Classification of Diseases, Ninth Revision, Clinical Modification (ICD-9-CM) diagnosis codes. **Table S3.** Baseline characteristics of people receiving second dose in recommended or delayed dosing interval by the brand of vaccine after the propensity score weighting. **Table S4.** Primary cause of unscheduled hospitalization (according to ICD-9-CM chapters) of people receiving second dose in recommended or delayed dosing interval by the type of vaccine. **Table S5.** Number of cases, incidence rates of all-cause mortality, emergency department visits, and unscheduled hospitalizations after second dose of BNT162b2 and CoronaVac by age. **Table S6.** Number of cases, incidence rates of all-cause mortality, emergency department visits, and unscheduled hospitalizations after second dose of BNT162b2 and CoronaVac in recommended, slightly delayed and delayed group. **Table S7.** Number of cases, incidence rates of all-cause mortality, emergency department visits, and unscheduled hospitalizations after excluding COVID-related outcomes or physical injuries. **Table S8.** Number of cases, incidence rates of all-cause mortality, emergency department visits, and unscheduled hospitalizations after including infinite delayers who delayed second dose for more than three months to delayed group. **Table S9.** Number of cases, incidence rates of all-cause mortality, emergency department visits, and unscheduled hospitalizations by using doubly robust method with inverse-probability-weighted regression-adjustment combination. **Fig. S1.** Distribution of second dose interval of BNT162b2 and CoronaVac recipients. **Fig. S2.** Distribution of propensity score density by the brand of vaccine before and after weighting.

## Data Availability

The data that support the findings of this study were extracted from the Hospital Authority database in Hong Kong. Restrictions apply to the availability of these data, which were used under license for this study. Data sharing is prohibited by the Hospital Authority. All the analysis codes that support the findings are available from the corresponding author upon reasonable requests.

## Abbreviations

AZD1222: ChAdOx1 nCoV-19 vaccine
CI: Confidence interval
CoronaVac: Inactivated SARS-CoV-2 vaccine
COVID-19: Coronavirus disease 2019
ED: Emergency department
HR: Hazard ratio
ICD-9-CM: International Classification of Diseases, Ninth Revision, Clinical Modification
IPTW: Inverse probability of treatment weights
IQR: Interquartile range
NSAIDs: Non-steroidal anti-inflammatory drugs
SD: Standard deviation
SMD: Standardized mean difference
