# Supplementary material for: Impact of a delayed second dose of mRNA vaccine (BNT162b2) and inactivated SARS-CoV-2 vaccine (CoronaVac) on risks of all-cause mortality, emergency department visit, and unscheduled hospitalization
Source: BMC Med. 2022 Mar 17;20:119. doi: 10.1186/s12916-022-02321-4 (PMC8926447; doi:10.1186/s12916-022-02321-4)

**Additional file 1 of**

**Impact of a delayed second dose of mRNA vaccine (BNT162b2) and inactivated** **SARS-CoV-2 vaccine (CoronaVac) on risks of all-cause mortality, emergency department visit, unscheduled hospitalization**

Carlos King Ho Wong^1,2,3*^ PhD, Xi Xiong^1*^ MSc, Kristy Tsz Kwan Lau^1^ MSc, Celine Sze Ling Chui^3,4,5^ PhD, Francisco Tsz Tsun Lai^1,3^ PhD, Xue Li^1,3,6^ PhD, Esther Wai Yin Chan^1,3^ PhD, Eric Yuk Fai Wan^1,2,3^ PhD, Ivan Chi Ho Au^1^ BSc, Benjamin John Cowling^3,5,7^ PhD, Cheuk Kwong Lee ^8^ PhD, Ian Chi Kei Wong^1,3,9^ PhD

*These first authors contributed equally to this article.

**Table of Contents**

[Table S1. Vaccination program priority groups rollout schedule in Hong Kong 2](#_Toc97113879)

[Table S2. Disease diagnosis defined by International Classification of Diseases, Ninth Revision, Clinical Modification (ICD-9-CM) diagnosis codes. 3](#_Toc97113880)

[Table S3. Baseline characteristics of people receiving second dose in recommended or delayed dosing interval by the brand of vaccine after the propensity score weighting 5](#_Toc97113881)

[Table S4. Primary cause of unscheduled hospitalization (according to ICD-9-CM chapters) of people receiving second dose in recommended or delayed dosing interval by the type of vaccine 6](#_Toc97113882)

[Table S5. Number of cases, incidence rates of all-cause mortality, emergency department visits, and unscheduled hospitalizations after second dose of BNT162b2 and CoronaVac by age. 8](#_Toc97113883)

[Table S6. Number of cases, incidence rates of all-cause mortality, emergency department visits, and unscheduled hospitalizations after second dose of BNT162b2 and CoronaVac in recommended, slightly delayed and delayed group. 9](#_Toc97113884)

[Table S7. Number of cases, incidence rates of emergency department visit, unscheduled hospitalization, and mortality after second dose excluding COVID-related outcomes or physical injuries. 10](#_Toc97113885)

[Table S8. Number of cases, incidence rates of emergency department visit, unscheduled hospitalization, and mortality after including infinite delayers who delayed second dose for more than three months to delayed group. 11](#_Toc97113886)

[Table S9. Sensitivity analysis by using doubly robust method with inverse-probability-weighted regression-adjustment combination. 12](#_Toc97113887)

[Figure S1. Distribution of second dose interval of BNT162b2 and CoronaVac recipients. 13](#_Toc97113888)

[Figure S2. Distribution of propensity score density by the brand of vaccine before and after weighting. 14](#_Toc97113889)

# Table S1. Vaccination program priority groups rollout schedule in Hong Kong

| Order of expansion | Date of rollout | Vaccination group |
| --- | --- | --- |
| First | Feb 26, 2021 | - Healthcare workers and staff involved in anti-epidemic work - Persons aged 60 or above and a maximum of 2 carers accompanying elderly people aged above 70 - Residents and staff of residential care homes for the elderly and persons with disabilities - People providing essential public services - People providing cross-boundary transportation or working at control points and ports |
| Second | Mar 8, 2021 | - Staff of food and beverages premises, markets, supermarkets, convenience stores, couriers and takeaway delivery - Staff of local public transport service operators - Registered construction workers - Staff of property management - Teachers and school staff - Staff in the tourism industry - Staff of scheduled premises under the Prevention and Control of Disease |
| Third | Mar 16, 2021 | - People aged between 30 and 59 - Students aged 16 or above studying outside Hong Kong - Domestic helpers |
| Four | Apr 15, 2021 | - People aged 16 to 29 (18 for person receiving CoronaVac) |
| Five | Jun 14, 2021 | - People aged 12 to 15 for BNT162b2 |

# Table S2. Disease diagnosis defined by International Classification of Diseases, Ninth Revision, Clinical Modification (ICD-9-CM) diagnosis codes.

| **Disease diagnosis** | **ICD-9 codes** |
| --- | --- |
| **Infectious And Parasitic Diseases** | 001-139 |
| Colitis, enteritis, and gastroenteritis of presumed infectious origin | 009.1 |
| **Neoplasms** | 140-239 |
| Malignancy | 140-159, 180-189, 170-172, 174-176, 179.X, 160-165, 190-195, 200-208 |
| Metastatic solid tumour | 196-199 |
| **Endocrine, Nutritional And Metabolic Diseases, And Immunity Disorders** | 240-279 |
| Diabetes mellitus | 250.X |
| Type 2 Diabetes | 250.00, 250.02, 250.10, 250.12, 250.20, 250.22, 250.30, 250.32, 250.40, 250.42, 250.50, 250.52, 250.60, 250.62, 250.70, 250.72, 250.80, 250.82, 250.90, 250.92 |
| Diabetes without chronic complication | 250.1-250.3, 250.7X |
| Diabetes with chronic complication | 250.4-250.6 |
| End-stage renal disease | 250.3X, 585.X, 586 |
| Severe hypoglycemia | 250.3X, 250.8X, 251.0-251.2, 270.3, 775.0, 775.6, 962.3 |
| Nephropathy | 250.40-250.43, 581-585.9, 249.4X, 791.0 |
| **Diseases Of The Blood And Blood-Forming Organs** | 280-289 |
| **Mental Disorders** | 290-319 |
| Psychosis | 291.1, 292-319 |
| **Diseases Of The Nervous System And Sense Organs** | 320-389 |
| **Diseases Of The Circulatory System** | 390-459 |
| Congestive heart failure | 398.91, 402.01, 402.11, 402.91, 404.01, 404.03, 404.11, 404.13, 404.91, 404.93, 428.X |
| Hypertension | 401.X, 402.X, 403.X, 404.X, 405.X |
| Coronary heart disease | 410.X, 411.X, 412.X, 413.X, 414.X |
| Myocardial infarction | 410 |
| Other chronic ischemic heart disease | 411-414.9 |
| Atrial fibrillation | 427.31 |
| Heart failure | 428.X |
| Stroke (Cerebrovascular disease) | 430-438.9 |
| Ischemic stroke | 433.01, 433.11, 433.21, 433.31, 433.81, 433.91, 434-436, 437.0X, 437.1X |
| Transient ischemic attack | 435.X |
| **Diseases Of The Respiratory System** | 460-519 |
| Pneumonia, organism unspecified | 486.X |
| Lung disease | 490-496 |
| Chronic obstructive pulmonary disease | 490-496, 500-505, 506.4X |
| COVID-19 comfirmed cases | 519.8x |
| **Diseases Of The Digestive System** | 520-579 |
| Ulcers | 531-534 |
| Peptic ulcer | 531.0-534.91 |
| Unspecified gastritis and gastroduodenitis | 535.50 |
| Other and unspecified noninfectious gastroenteritis and colitis | 558.9 |
| Liver disease | 570-573.9 |
| **Diseases Of The Genitourinary System** | 580-629 |
| Chronic renal failure | 582.X, 585.X, 586.X, 588.X, 583.0-583.2, 583.4X, 583.6-583.7 |
| Chronic kidney disease | 585.X, 586.X |
| Calculus of ureter | 592.1 |
| Other specified disorders of kidney and ureter | 593.83 |
| Urinary tract infection, site not specified | 599.0 |
| Hematuria | 599.7 |
| Excessive or frequent menstruation | 626.2 |
| **Diseases Of The Skin And Subcutaneous Tissue** | 680-709 |
| Cellulitis and abscess of leg, except foot | 682.6 |
| Sebaceous cyst | 706.2 |
| **Diseases Of The Musculoskeletal System And Connective Tissue** | 710-739 |
| Rheumatoid arthritis and other inflammatory polyarthropathies | 710.0X, 710.1X, 710.4X, 714.0-714.2, 714.81, 725.X |
| **Symptoms, Signs, And Ill-Defined Conditions** | 780-799 |
| Symptoms involving skin and other integumentary tissue | 782.0 |
| Syncope and collapse | 780.2 |
| Dizziness and giddiness | 780.4 |
| Fever and other physiologic disturbances of temperature regulation | 780.6 |
| Palpitations | 785.1 |
| Chest pain | 786.50 |
| Other symptoms involving abdomen and pelvis | 789.00 |
| Abdominal pain, epigastric | 789.06 |
| Elevated prostate specific antigen | 790.93 |
| **Injury And Poisoning** | 800-999 |
| Physical injuries | 800-904, 910-959, 980-985, 987-995 |
| Other closed fractures of distal end of radius (alone) | 813.42 |
| Intracranial injury of other and unspecified nature without mention of open intracranial wound, with no loss of consciousness | 854.01 |
| Open wound(s) (multiple) of unspecified site(s), without mention of complication | 879.8 |
| **Supplementary Classification Of Factors Influencing Health Status And Contact With Health Services** | V01-V82 |
| Observation for unspecified suspected condition | V71.9 |

# Table S3. Baseline characteristics of people receiving second dose in recommended or delayed dosing interval by the brand of vaccine after the propensity score weighting

|  | After weighting | | | | | |
| --- | --- | --- | --- | --- | --- | --- |
|  | BNT162b2 | | CoronoVac | | BNT162b2 | CoronoVac |
| Baseline characteristics | Recommended (N = 401,473) | Delayed (N = 16,024) | Recommended (N = 253,427) | Delayed (N = 100,856) |  |  |
|  | Mean±SD / % | Mean±SD / % | Mean±SD / % | Mean±SD / % | SMD | SMD |
| Age, years | 47.3±14.9 | 47.1±15.1 | 54.4±13.9 | 54.2±14.1 | 0.02 | 0.02 |
| 16-44 | 44.9% | 46.2% | 24.1% | 24.9% | 0.03 | 0.02 |
| 45-64 | 41.2% | 39.8% | 50.8% | 50.3% |  |  |
| ≥65 | 13.9% | 14.0% | 25.1% | 24.8% |  |  |
| Sex |  |  |  |  | 0.01 | 0.01 |
| Male | 48.8% | 48.5% | 52.3% | 52.0% |  |  |
| Female | 51.2% | 51.5% | 47.7% | 48.0% |  |  |
| Region |  |  |  |  | 0.01 | 0.01 |
| Hong Kong Island | 21.4% | 20.9% | 13.4% | 13.4% |  |  |
| Kowloon | 27.1% | 26.9% | 31.1% | 31.8% |  |  |
| New Territories | 51.3% | 52.0% | 55.3% | 54.7% |  |  |
| Unknown | 0.2% | 0.2% | 0.2% | 0.2% |  |  |
| Dosing interval, days | 22.1±2.0 | 34.1±7.8 | 28.0±0.0 | 31.7±6.6 | NA | NA |
| Vaccination site |  |  |  |  | 0.01 | 0.01 |
| Community vaccination centres | 99.5% | 99.5% | 62.6% | 62.1% |  |  |
| Clinics | 0.0% | 0.0% | 36.0% | 36.4% |  |  |
| Others | 0.5% | 0.5% | 1.4% | 1.5% |  |  |
| ED visit between first and second doses | 2.3% | 3.0% | 2.9% | 3.1% | 0.01 | 0.01 |
| Unscheduled hospitalization between first and second dose | 0.4% | 0.9% | 0.6% | 0.8% | 0.01 | 0.01 |
| COVID-19 survivor | 0.1% | 0.1% | 0.1% | 0.1% | 0.00 | 0.00 |
| Pre-existing comorbidities |  |  |  |  |  |  |
| Charlson's Index | 1.4±1.4 | 1.4±1.5 | 2.1±1.5 | 2.0±1.5 | 0.01 | 0.02 |
| 0 | 35.4% | 37.0% | 16.6% | 17.6% | 0.03 | 0.03 |
| 1-2 | 41.0% | 39.8% | 44.5% | 44.7% |  |  |
| ≥3 | 23.6% | 23.2% | 38.9% | 37.8% |  |  |
| Myocardial infarction | 0.1% | 0.1% | 0.2% | 0.1% | 0.00 | 0.01 |
| Ischemic stroke | 0.2% | 0.3% | 0.4% | 0.4% | 0.02 | 0.00 |
| Transient ischemic attack | 0.1% | 0.2% | 0.2% | 0.2% | 0.01 | 0.00 |
| Congestive heart failure | 0.1% | 0.1% | 0.2% | 0.2% | 0.00 | 0.00 |
| Cerebrovascular disease | 0.7% | 1.0% | 1.3% | 1.3% | 0.03 | 0.00 |
| Chronic obstructive pulmonary disease | 0.8% | 1.0% | 1.0% | 1.0% | 0.02 | 0.01 |
| Diabetes without chronic complication | 4.7% | 4.2% | 7.5% | 7.0% | 0.03 | 0.02 |
| Diabetes with chronic complication | 0.1% | 0.2% | 0.2% | 0.2% | 0.00 | 0.00 |
| Chronic renal failure | 0.2% | 0.2% | 0.4% | 0.4% | 0.01 | 0.00 |
| Ulcers | 0.3% | 0.3% | 0.5% | 0.6% | 0.00 | 0.00 |
| Rheumatoid arthritis and other inflammatory polyarthropathies | 0.1% | 0.1% | 0.1% | 0.1% | 0.01 | 0.01 |
| Malignancy | 0.8% | 0.8% | 0.9% | 1.0% | 0.01 | 0.01 |
| Metastatic solid tumour | 0.1% | 0.1% | 0.1% | 0.1% | 0.02 | 0.01 |
| Drug history |  |  |  |  |  |  |
| Renin-angiotensin-system agents | 6.9% | 6.6% | 10.2% | 9.9% | 0.01 | 0.01 |
| Beta blockers | 3.9% | 3.9% | 5.8% | 5.4% | 0.00 | 0.02 |
| Calcium channel blockers | 10.3% | 9.2% | 15.9% | 15.1% | 0.04 | 0.02 |
| Lipid lowering agents | 10.4% | 10.8% | 15.5% | 14.9% | 0.01 | 0.02 |
| Antidiabetic drugs | 4.9% | 4.4% | 7.6% | 7.2% | 0.02 | 0.02 |
| Antiplatelets | 3.0% | 3.4% | 4.9% | 4.7% | 0.02 | 0.01 |
| Antidepressants | 3.0% | 3.0% | 3.3% | 3.1% | 0.00 | 0.01 |
| NSAIDs | 6.3% | 7.5% | 6.2% | 6.4% | 0.05 | 0.01 |
| Drugs for gout | 1.0% | 1.1% | 1.6% | 1.5% | 0.01 | 0.00 |
| Antiepileptic drugs | 1.2% | 1.5% | 1.1% | 1.0% | 0.02 | 0.01 |
| Antiviral drugs | 1.0% | 1.0% | 1.2% | 1.1% | 0.00 | 0.00 |
| Antibacterial drugs | 2.9% | 3.6% | 2.8% | 2.9% | 0.04 | 0.01 |

Note: ED = emergency department; SD = standard deviation; SMD = absolute standardized mean difference; NA = Not available.

Received second dose 21-28 days (BNT162b2) or 14-28 days (CoronaVac) after first dose was considered within recommended interval; Received second dose 28 days after first dose was regarded as delayed second dose.

# Table S4. Primary cause of unscheduled hospitalization (according to ICD-9-CM chapters) of people receiving second dose in recommended or delayed dosing interval by the type of vaccine

| **Primary cause of hospitalization** | **BNT162b2** | | **CoronaVac** | |
| --- | --- | --- | --- | --- |
|  | **Recommended  (N=2,929)** | **Delayed  (N=124)** | **Recommended  (N=2,068)** | **Delayed  (N=781)** |
| **Infectious And Parasitic Diseases** | 1.84%(54) | 1.61%(2) | 2.47%(51) | 2.43%(19) |
| Colitis, enteritis, and gastroenteritis of presumed infectious origin | 0.89%(26) | 1.61%(2) | 1.11%(23) | 0.64%(5) |
| **Neoplasms** | 1.26%(37) | 0.81%(1) | 1.35%(28) | 1.66%(13) |
| **Endocrine, Nutritional And Metabolic Diseases, And Immunity Disorders** | 1.95%(57) | 2.42%(3) | 2.08%(43) | 3.33%(26) |
| Diabetes mellitus | 0.72%(21) | 0.81%(1) | 0.68%(14) | 1.15%(9) |
| Type 2 Diabetes | 0.72%(21) | 0.81%(1) | 0.68%(14) | 1.15%(9) |
| End-stage renal disease | 0.10%(3) | 0.81%(1) | 0.15%(3) | 0.13%(1) |
| Severe hypoglycemia | 0.51%(15) | 0%(0) | 0.44%(9) | 0.26%(2) |
| Nephropathy | 0.31%(9) | 0%(0) | 0.15%(3) | 0.26%(2) |
| **Diseases Of The Blood And Blood-Forming Organs** | 0.65%(19) | 0.81%(1) | 0.58%(12) | 0.38%(3) |
| **Mental Disorders** | 2.29%(67) | 4.03%(5) | 2.13%(44) | 2.82%(22) |
| Psychosis | 2.25%(66) | 4.03%(5) | 2.08%(43) | 2.69%(21) |
| **Diseases Of The Nervous System And Sense Organs** | 1.98%(58) | 0%(0) | 1.64%(34) | 1.54%(12) |
| **Diseases Of The Circulatory System** | 8.13%(238) | 11.29%(14) | 10.78%(223) | 8.96%(70) |
| Hypertension | 2.90%(85) | 3.23%(4) | 2.85%(59) | 3.33%(26) |
| Coronary heart disease | 1.37%(40) | 0.81%(1) | 1.74%(36) | 1.28%(10) |
| Myocardial infarction | 0.99%(29) | 0%(0) | 0.77%(16) | 0.51%(4) |
| Other chronic ischemic heart disease | 0.38%(11) | 0.81%(1) | 0.97%(20) | 0.77%(6) |
| Atrial fibrillation | 0.34%(10) | 0.81%(1) | 0.53%(11) | 0.51%(4) |
| Heart failure | 0.14%(4) | 0%(0) | 0.39%(8) | 0.26%(2) |
| Stroke | 1.95%(57) | 2.42%(3) | 3.43%(71) | 2.56%(20) |
| **Diseases Of The Respiratory System** | 2.22%(65) | 2.42%(3) | 3.38%(70) | 3.33%(26) |
| Pneumonia, organism unspecified | 0.68%(20) | 0.81%(1) | 0.97%(20) | 0.90%(7) |
| Lung disease | 0.58%(17) | 0.81%(1) | 0.87%(18) | 0.77%(6) |
| **Diseases Of The Digestive System** | 8.02%(235) | 5.65%(7) | 8.61%(178) | 9.09%(71) |
| Peptic ulcer | 0.44%(13) | 0%(0) | 0.58%(12) | 0.64%(5) |
| Unspecified gastritis and gastroduodenitis | 0.99%(29) | 1.61%(2) | 0.92%(19) | 1.02%(8) |
| Other and unspecified noninfectious gastroenteritis and colitis | 0.38%(11) | 1.61%(2) | 0.53%(11) | 0.51%(4) |
| Liver disease | 0.38%(11) | 0.81%(1) | 0.63%(13) | 0.64%(5) |
| **Diseases Of The Genitourinary System** | 6.79%(199) | 6.45%(8) | 6.53%(135) | 6.66%(52) |
| Chronic kidney disease | 0.10%(3) | 0.81%(1) | 0.15%(3) | 0.13%(1) |
| Calculus of ureter | 0.44%(13) | 0%(0) | 0.48%(10) | 0%(0) |
| Other specified disorders of kidney and ureter | 1.33%(39) | 0.81%(1) | 0.87%(18) | 1.66%(13) |
| Urinary tract infection, site not specified | 0.82%(24) | 1.61%(2) | 0.82%(17) | 1.41%(11) |
| Hematuria | 0.24%(7) | 0%(0) | 1.11%(23) | 0.90%(7) |
| Excessive or frequent menstruation | 0.55%(16) | 0.81%(1) | 0.58%(12) | 0.77%(6) |
| **Diseases Of The Skin And Subcutaneous Tissue** | 3.07%(90) | 3.23%(4) | 3.29%(68) | 3.20%(25) |
| Cellulitis and abscess of leg, except foot | 0.68%(20) | 0.81%(1) | 0.82%(17) | 0.51%(4) |
| Sebaceous cyst | 0.20%(6) | 0%(0) | 0.15%(3) | 0.13%(1) |
| **Diseases Of The Musculoskeletal System And Connective Tissue** | 4.17%(122) | 1.61%(2) | 6.00%(124) | 5.76%(45) |
| **Symptoms, Signs, And Ill-Defined Conditions** | 36.16%(1,059) | 41.94%(52) | 31.43%(650) | 32.65%(255) |
| Symptoms involving skin and other integumentary tissue | 1.57%(46) | 4.03%(5) | 1.35%(28) | 1.41%(11) |
| Syncope and collapse | 1.91%(56) | 0.81%(1) | 1.21%(25) | 1.15%(9) |
| Dizziness and giddiness | 3.45%(101) | 4.84%(6) | 5.27%(109) | 4.87%(38) |
| Fever and other physiologic disturbances of temperature regulation | 3.28%(96) | 0.81%(1) | 1.60%(33) | 1.41%(11) |
| Palpitations | 2.56%(75) | 4.84%(6) | 1.60%(33) | 1.54%(12) |
| Chest pain | 8.06%(236) | 9.68%(12) | 6.19%(128) | 6.66%(52) |
| Other symptoms involving abdomen and pelvis | 3.72%(109) | 3.23%(4) | 3.82%(79) | 4.10%(32) |
| Abdominal pain, epigastric | 1.33%(39) | 0.81%(1) | 1.45%(30) | 1.92%(15) |
| Elevated prostate specific antigen | 0.07%(2) | 0%(0) | 0.05%(1) | 0%(0) |
| **Injury And Poisoning** | 13.38%(392) | 11.29%(14) | 14.12%(292) | 12.68%(99) |
| Other closed fractures of distal end of radius (alone) | 0.79%(23) | 1.61%(2) | 0.87%(18) | 0.77%(6) |
| Intracranial injury of other and unspecified nature without mention of open intracranial wound, with no loss of consciousness | 0.85%(25) | 1.61%(2) | 1.11%(23) | 1.66%(13) |
| Open wound(s) (multiple) of unspecified site(s), without mention of complication | 1.06%(31) | 0%(0) | 1.6%(33) | 1.02%(8) |
| **Supplementary Classification Of Factors Influencing Health Status And Contact With Health Services** | 1.71%(50) | 2.42%(3) | 1.16%(24) | 1.41%(11) |
| Observation for unspecified suspected condition | 1.26%(37) | 1.61%(2) | 0.87%(18) | 1.28%(10) |
| **Unknown** | 6.40% (187) | 0.60% (5) | 4.40% (92) | 3.50% (32) |

# Table S5. Number of cases, incidence rates of all-cause mortality, emergency department visits, and unscheduled hospitalizations after second dose of BNT162b2 and CoronaVac by age.

|  | Before weighting | | | | | | | | | | After weighting | | |
| --- | --- | --- | --- | --- | --- | --- | --- | --- | --- | --- | --- | --- | --- |
|  | Recommended (N=654,900) | | | | | Delayed (N=116,880) | | | | | Delayed vs Recommended interval | | |
| Event | Cumulative incidence | | Crude incidence rate  (Events / 10,000 person-days) | | | Cumulative incidence | | Crude incidence rate  (Events / 10,000 person-days) | | | HR | 95% CI | P-value |
|  | Cases with event | Rate | Estimate | 95% CI | Person-days | Cases with event | Rate | Estimate | 95% CI | Person-days |  |  |  |
| **Mortality after second dose** | | | | |  |  |  |  |  |  |  |  |  |
| **BNT162b2** | 15 | 0.0037% | 0.0133 | (0.01, 0.02) | 11,241,070 | 2 | 0.0125% | 0.0446 | (0.01, 0.16) | 448,656 | 4.438 | (0.951, 20.701) | 0.058 |
| 16-44 | 6 | 0.0034% | 0.0120 | (0.00, 0.03) | 5,001,649 | 1 | 0.0111% | 0.0397 | (0.00, 0.22) | 251,766 | NA | NA | NA |
| 45-64 | 5 | 0.0030% | 0.0107 | (0.00, 0.03) | 4,654,617 | 0 | NA | NA | NA | NA | NA | NA | NA |
| ≥65 | 4 | 0.0071% | 0.0252 | (0.01, 0.06) | 1,584,804 | 1 | 0.0696% | 0.2487 | (0.01, 1.39) | 40,202 | NA | NA | NA |
| **CoronaVac** | 16 | 0.0063% | 0.0225 | (0.01, 0.04) | 7,095,767 | 7 | 0.0069% | 0.0248 | (0.01, 0.05) | 2,823,889 | 1.185 | (0.478, 2.937) | 0.714 |
| 16-44 | 1 | 0.0017% | 0.0061 | (0.00, 0.03) | 1,643,141 | 1 | 0.0037% | 0.0134 | (0.00, 0.07) | 747,400 | NA | NA | NA |
| 45-64 | 9 | 0.0070% | 0.0250 | (0.01, 0.05) | 3,597,295 | 3 | 0.0059% | 0.0212 | (0.00, 0.06) | 1,414,399 | 1.028 | (0.278, 3.810) | 0.966 |
| ≥65 | 6 | 0.0091% | 0.0323 | (0.01, 0.07) | 1,855,331 | 3 | 0.0127% | 0.0453 | (0.01, 0.13) | 662,090 | 1.185 | (0.279, 5.024) | 0.818 |
|  |  |  |  |  |  |  |  |  |  |  |  |  |  |
| **ED visit after second dose** | | | | | |  |  |  |  |  |  |  |  |
| **BNT162b2** | 13,818 | 3.44% | 12.55 | (12.34, 12.76) | 11,011,002 | 635 | 3.96% | 14.49 | (13.38, 15.66) | 438,314 | 1.037 | (0.951, 1.130) | 0.411 |
| 16-44 | 7,404 | 4.14% | 15.19 | (14.85, 15.54) | 4,874,381 | 376 | 4.18% | 15.32 | (13.81, 16.94) | 245,504 | 0.945 | (0.848, 1.052) | 0.302 |
| 45-64 | 5,164 | 3.11% | 11.30 | (10.99, 11.61) | 4,570,059 | 216 | 3.86% | 14.09 | (12.27, 16.09) | 153,351 | 1.151 | (0.997, 1.330) | 0.055 |
| ≥65 | 1,250 | 2.21% | 7.98 | (7.54, 8.43) | 1,566,563 | 43 | 2.99% | 10.90 | (7.89, 14.68) | 39,459 | 1.102 | (0.780, 1.558) | 0.580 |
| **CoronaVac** | 7,856 | 3.10% | 11.26 | (11.01, 11.51) | 6,977,752 | 3,112 | 3.09% | 11.21 | (10.82, 11.61) | 2,776,922 | 0.966 | (0.926, 1.008) | 0.113 |
| 16-44 | 2,229 | 3.80% | 13.85 | (13.28, 14.43) | 1,609,685 | 956 | 3.58% | 13.04 | (12.23, 13.90) | 733,039 | 0.937 | (0.867, 1.012) | 0.097 |
| 45-64 | 3,990 | 3.11% | 11.28 | (10.93, 11.64) | 3,537,061 | 1,570 | 3.11% | 11.29 | (10.74, 11.86) | 1,390,371 | 0.965 | (0.909, 1.025) | 0.243 |
| ≥65 | 1,637 | 2.47% | 8.94 | (8.51, 9.38) | 1,831,006 | 586 | 2.48% | 8.97 | (8.26, 9.72) | 653,512 | 1.002 | (0.909, 1.105) | 0.963 |
|  |  |  |  |  |  |  |  |  |  |  |  |  |  |
| **Unscheduled hospitalization after second dose** | | | | | |  |  |  |  |  |  |  |  |
| **BNT162b2** | 2,929 | 0.73% | 2.62 | (2.52, 2.71) | 11,195,142 | 124 | 0.77% | 2.78 | (2.31, 3.31) | 446,704 | 1.054 | (0.867, 1.281) | 0.597 |
| 16-44 | 1,270 | 0.71% | 2.55 | (2.41, 2.69) | 4,980,781 | 58 | 0.65% | 2.31 | (1.76, 2.99) | 250,816 | 0.893 | (0.679, 1.175) | 0.418 |
| 45-64 | 1,212 | 0.73% | 2.61 | (2.47, 2.77) | 4,635,614 | 45 | 0.80% | 2.88 | (2.10, 3.86) | 156,015 | 1.036 | (0.759, 1.413) | 0.825 |
| ≥65 | 447 | 0.79% | 2.83 | (2.57, 3.11) | 1,578,748 | 21 | 1.46% | 5.27 | (3.26, 8.05) | 39,873 | 1.584 | (0.972, 2.583) | 0.065 |
| **CoronaVac** | 2,068 | 0.82% | 2.93 | (2.80, 3.06) | 7,065,542 | 781 | 0.77% | 2.78 | (2.59, 2.98) | 2,812,393 | 0.956 | (0.878, 1.040) | 0.294 |
| 16-44 | 413 | 0.70% | 2.52 | (2.29, 2.78) | 1,637,153 | 151 | 0.57% | 2.03 | (1.72, 2.38) | 745,109 | 0.800 | (0.661, 0.967) | 0.021* |
| 45-64 | 965 | 0.75% | 2.69 | (2.53, 2.87) | 3,582,953 | 390 | 0.77% | 2.77 | (2.50, 3.06) | 1,408,596 | 1.024 | (0.907, 1.155) | 0.703 |
| ≥65 | 690 | 1.04% | 3.74 | (3.47, 4.03) | 1,845,436 | 240 | 1.01% | 3.64 | (3.20, 4.13) | 658,689 | 0.968 | (0.832, 1.126) | 0.669 |

Note: HR = Hazard ratio; CI = Confidence interval; NA = Not available;

HRs were not estimated when the number of events in one of the groups was ≤1.

* No association as it did not reach Bonferroni-corrected significance level.

† HR >1 (or <1) indicates persons delaying second does had higher risk (or lower risk) of outcome events after second dose, compared to persons receiving second dose within recommended interval.

# Table S6. Number of cases, incidence rates of all-cause mortality, emergency department visits, and unscheduled hospitalizations after second dose of BNT162b2 and CoronaVac in recommended, slightly delayed and delayed group.

| Subgroups | Cumulative incidence (Events / 10,000 doses) | | Crude incidence rate  (Events / 10,000 person-years) | | HR* | 95% CI | P-value |
| --- | --- | --- | --- | --- | --- | --- | --- |
|  | Cases with event | Rate | Estimate | 95% CI |  |  |  |
| **Mortality after second dose** | |  |  |  |  |  |  |
| **BNT162b2** |  |  |  |  |  |  |  |
| Recommended | 15 | 0.0037% | 0.0133 | (0.01, 0.02) | Reference | | |
| Slightly delayed | 2 | 0.0136% | 0.0487 | (0.01, 0.18) | 4.612 | (0.948, 22.429) | 0.058 |
| More delayed | 0 | 0.0000% | 0.0000 | NA | NA | NA | NA |
| **CoronaVac** |  |  |  |  |  |  |  |
| Recommended | 16 | 0.0063% | 0.0225 | (0.01, 0.04) | Reference | | |
| Slightly delayed | 7 | 0.0070% | 0.0251 | (0.01, 0.05) | 1.213 | (0.489, 3.005) | 0.677 |
| More delayed | 0 | 0.0000% | 0.0000 | NA | NA | NA | NA |
|  |  |  |  |  |  |  |  |
| **ED visit after second dose** | | |  |  |  |  |  |
| **BNT162b2** |  |  |  |  |  |  |  |
| Recommended | 13,818 | 3.44% | 12.55 | (12.34, 12.76) | Reference | | |
| Slightly delayed | 572 | 3.90% | 14.24 | (13.10, 15.45) | 1.019 | (0.932, 1.115) | 0.674 |
| More delayed | 63 | 4.69% | 17.22 | (13.23, 22.03) | 1.087 | (0.800, 1.478) | 0.594 |
| **CoronaVac** |  |  |  |  |  |  |  |
| Recommended | 7,856 | 3.10% | 11.26 | (11.01, 11.51) | Reference | | |
| Slightly delayed | 3,063 | 3.07% | 11.15 | (10.76, 11.55) | 0.962 | (0.921, 1.004) | 0.074 |
| More delayed | 49 | 4.38% | 16.05 | (11.87, 21.22) | 1.480 | (1.026, 2.136) | 0.036* |
|  |  |  |  |  |  |  |  |
| **Unscheduled hospitalization after second dose** | | | |  |  |  |  |
| **BNT162b2** |  |  |  |  |  |  |  |
| Recommended | 2,929 | 0.73% | 2.62 | (2.52, 2.71) | Reference | | |
| Slightly delayed | 103 | 0.70% | 2.52 | (2.05, 3.05) | 0.963 | (0.780, 1.190) | 0.730 |
| More delayed | 21 | 1.56% | 5.63 | (3.49, 8.61) | 1.878 | (1.112, 3.171) | 0.018* |
| **CoronaVac** |  |  |  |  |  |  |  |
| Recommended | 2,068 | 0.82% | 2.93 | (2.80, 3.06) | Reference | | |
| Slightly delayed | 768 | 0.77% | 2.76 | (2.57, 2.96) | 0.953 | (0.876, 1.038) | 0.272 |
| More delayed | 13 | 1.16% | 4.18 | (2.22, 7.14) | 1.164 | (0.564, 2.402) | 0.682 |

Note: HR = Hazard ratio; CI = Confidence interval; NA = Not available

* No association as it did not reach Bonferroni-corrected significance level.

† HR >1 (or <1) indicates persons delaying second does had higher risk (or lower risk) of outcome events after second dose, compared to persons receiving second dose within recommended interval.

# Table S7. Number of cases, incidence rates of emergency department visit, unscheduled hospitalization, and mortality after second dose excluding COVID-related outcomes or physical injuries.

|  | Before weighting | | | | | | | | | | After weighting | | | |
| --- | --- | --- | --- | --- | --- | --- | --- | --- | --- | --- | --- | --- | --- | --- |
|  | Recommended (N=654,900) | | | | | Delayed (N=116,880) | | | | | Delayed vs Recommended interval | | | |
| Event | Cumulative incidence | | Crude incidence rate  (Events / 10,000 person-days) | | | Cumulative incidence | | Crude incidence rate  (Events / 10,000 person-days) | | | HR | 95% CI | P-value |  |
|  | Cases with event | Rate | Estimate | 95% CI | Person-days | Cases with event | Rate | Estimate | 95% CI | Person-days |  |  |  |  |
| **Mortality after second dose** | | |  |  |  |  |  |  |  |  |  |  |  |  |
| **BNT162b2** | 15 | 0.0037% | 0.0133 | (0.01, 0.02) | 11,241,070 | 1 | 0.0062% | 0.0223 | (0.00, 0.12) | 448,666 | NA | NA | NA |  |
| **CoronaVac** | 16 | 0.0063% | 0.0225 | (0.01, 0.04) | 7,095,767 | 7 | 0.0069% | 0.0248 | (0.01, 0.05) | 2,823,889 | 1.185 | (0.478, 2.937) | 0.714 |  |
|  |  |  |  |  |  |  |  |  |  |  |  |  |  |  |
| **ED visit after second dose** | | | | |  |  |  |  |  |  |  |  |  |  |
| **BNT162b2** | 13,818 | 3.44% | 12.55 | (12.34, 12.76) | 11,011,002 | 635 | 3.96% | 14.49 | (13.38, 15.66) | 438,314 | 1.037 | (0.951, 1.130) | 0.411 |  |
| **CoronaVac** | 7,856 | 3.10% | 11.26 | (11.01, 11.51) | 6,977,752 | 3,112 | 3.09% | 11.21 | (10.82, 11.61) | 2,776,922 | 0.966 | (0.926, 1.008) | 0.113 |  |
|  |  |  |  |  |  |  |  |  |  |  |  |  |  |  |
| **Unscheduled hospitalization after second dose** | | | | |  |  |  |  |  |  |  |  |  |  |
| **BNT162b2** | 2,888 | 0.72% | 2.58 | (2.49, 2.68) | 11,196,018 | 124 | 0.77% | 2.78 | (2.31, 3.31) | 446,714 | 1.069 | (0.880, 1.299) | 0.502 |  |
| **CoronaVac** | 2,048 | 0.81% | 2.90 | (2.77, 3.03) | 7,065,876 | 775 | 0.77% | 2.76 | (2.56, 2.96) | 2,812,522 | 0.958 | (0.880, 1.043) | 0.324 |  |

Note: HR = Hazard ratio; CI = Confidence interval; NA = Not available

HRs were not estimated when the number of events in one of the groups was ≤1.

† HR >1 (or <1) indicates persons delaying second does had higher risk (or lower risk) of outcome events after second dose, compared to persons receiving second dose within recommended interval.

# Table S8. Number of cases, incidence rates of emergency department visit, unscheduled hospitalization, and mortality after including infinite delayers who delayed second dose for more than three months to delayed group.

|  | Before weighting | | | | | | | | | | | Before weighting | | | | | |
| --- | --- | --- | --- | --- | --- | --- | --- | --- | --- | --- | --- | --- | --- | --- | --- | --- | --- |
|  | Recommended (N=654,900) | | | | | Delayed (N=121,503) | | | | | | Delayed vs Recommended interval | | | | | |
| Event | Cumulative incidence | | Crude incidence rate  (Events / 10,000 person-days) | | | Cumulative incidence | | Crude incidence rate  (Events / 10,000 person-days) | | | | HR | | 95% CI | | P-value | |
|  | Cases with event | Rate | Estimate | 95% CI | Person-days | Cases with event | Rate | Estimate | 95% CI | Person-days |  | |  | |  | |  |
| **Mortality after second dose** | | | | | |  |  |  |  |  |  | |  | |  | |  |
| **BNT162b2** | 15 | 0.0037% | 0.0133 | (0.01, 0.02) | 11,241,070 | 2 | 0.0113% | 0.0404 | (0.00, 0.15) | 495,164 | 4.078 | | (0.889, 18.709) | | 0.071 | |  |
| **CoronaVac** | 16 | 0.0063% | 0.0225 | (0.01, 0.04) | 7,095,767 | 12 | 0.0116% | 0.0413 | (0.02, 0.07) | 2,906,759 | 1.662 | | (0.767, 3.602) | | 0.198 | |  |
|  |  |  |  |  |  |  |  |  |  |  |  | |  | |  | |  |
| **ED visit after second dose** | | | | | | |  |  |  |  |  | |  | |  | |  |
| **BNT162b2** | 13,818 | 3.44% | 12.55 | (12.34, 12.76) | 11,011,002 | 730 | 4.13% | 15.11 | (14.03, 16.25) | 483,184 | 1.074 | | (0.990, 1.166) | | 0.087 | |  |
| **CoronaVac** | 7,856 | 3.10% | 11.26 | (11.01, 11.51) | 6,977,752 | 3,317 | 3.20% | 11.61 | (11.22, 12.01) | 2,856,430 | 0.993 | | (0.953, 1.036) | | 0.749 | |  |
|  |  |  |  |  |  |  |  |  |  |  |  | |  | |  | |  |
| **Unscheduled hospitalization after second dose** | | | | | | |  |  |  |  |  | |  | |  | |  |
| **BNT162b2** | 2,929 | 0.73% | 2.62 | (2.52, 2.71) | 11,195,142 | 167 | 0.94% | 3.39 | (2.90, 3.95) | 492,481 | 1.193 | | (0.999, 1.424) | | 0.051 | |  |
| **CoronaVac** | 2,068 | 0.82% | 2.93 | (2.80, 3.06) | 7,065,542 | 904 | 0.87% | 3.12 | (2.92, 3.34) | 2,893,211 | 1.046 | | (0.966, 1.134) | | 0.269 | |  |

Note: HR = Hazard ratio; CI = Confidence interval

† HR >1 (or <1) indicates persons delaying second does had higher risk of emergency department visit after second dose, compared to persons receiving second dose within recommended interval.

# Table S9. Sensitivity analysis by using doubly robust method with inverse-probability-weighted regression-adjustment combination.

|  | Before weighting | | | | | | | | | | Doubly robust method | | |
| --- | --- | --- | --- | --- | --- | --- | --- | --- | --- | --- | --- | --- | --- |
|  | Recommended (N=654,900) | | | | | Delayed (N=116,880) | | | | | Delayed vs Recommended interval | | |
| Event | Cumulative incidence | | Crude incidence rate  (Events / 10,000 person-days) | | | Cumulative incidence | | Crude incidence rate  (Events / 10,000 person-days) | | | HR | 95% CI | P-value |
|  | Cases with event | Rate | Estimate | 95% CI | Person-days | Cases with event | Rate | Estimate | 95% CI | Person-days |  |  |  |
| **Mortality after second dose** | | |  |  |  |  |  |  |  |  |  |  |  |
| **BNT162b2** | 15 | 0.0037% | 0.0133 | (0.01, 0.02) | 11,241,070 | 2 | 0.0125% | 0.0446 | (0.01, 0.16) | 448,656 | 4.425 | (0.980, 19.979) | 0.053 |
| **CoronaVac** | 16 | 0.0063% | 0.0225 | (0.01, 0.04) | 7,095,767 | 7 | 0.0069% | 0.0248 | (0.01, 0.05) | 2,823,889 | 1.203 | (0.479, 3.021) | 0.694 |
|  |  |  |  |  |  |  |  |  |  |  |  |  |  |
| **ED visit after second dose** | |  |  |  |  |  |  |  |  |  |  |  |  |
| **BNT162b2** | 13,818 | 3.44% | 12.55 | (12.34, 12.76) | 11,011,002 | 635 | 3.96% | 14.49 | (13.38, 15.66) | 438,314 | 1.027 | (0.942, 1.120) | 0.541 |
| **CoronaVac** | 7,856 | 3.10% | 11.26 | (11.01, 11.51) | 6,977,752 | 3,112 | 3.09% | 11.21 | (10.82, 11.61) | 2,776,922 | 0.959 | (0.919, 1.001) | 0.053 |
|  |  |  |  |  |  |  |  |  |  |  |  |  |  |
| **Unscheduled hospitalization after second dose** | | | |  |  |  |  |  |  |  |  |  |  |
| **BNT162b2** | 2,929 | 0.73% | 2.62 | (2.52, 2.71) | 11,195,142 | 124 | 0.77% | 2.78 | (2.31, 3.31) | 446,704 | 1.039 | (0.853, 1.264) | 0.706 |
| **CoronaVac** | 2,068 | 0.82% | 2.93 | (2.80, 3.06) | 7,065,542 | 781 | 0.77% | 2.78 | (2.59, 2.98) | 2,812,393 | 0.955 | (0.877, 1.040) | 0.289 |

Note: HR = Hazard ratio; CI = Confidence interval

† HR >1 (or <1) indicates persons delaying second does had higher risk of emergency department visit after second dose, compared to persons receiving second dose within recommended interval.

# Figure S1. Distribution of second dose interval of BNT162b2 and CoronaVac recipients.


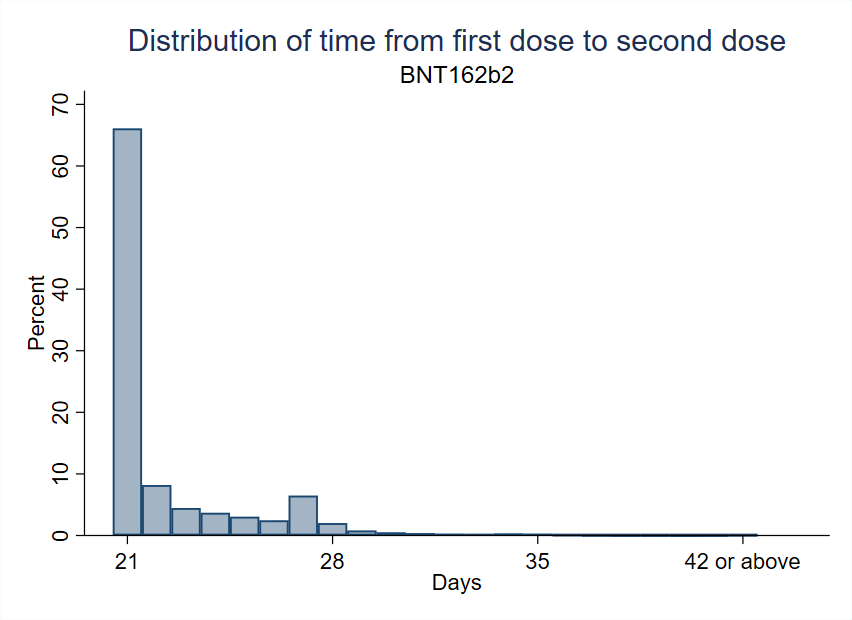

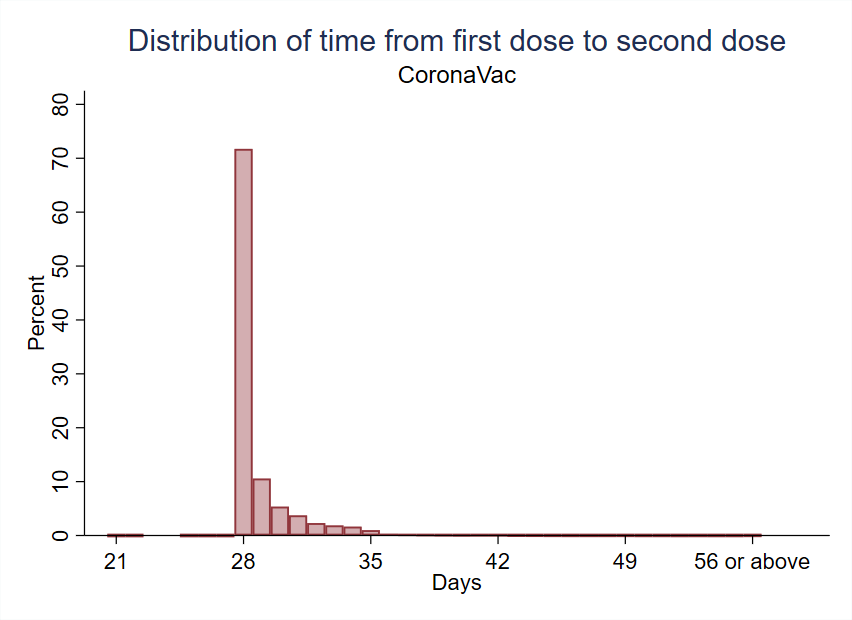


Mean dosing interval for BNT162b2 recipients is 22.6 days (Standard deviation [SD]: 3.5), ranging from 21 to 114 days.

Mean dosing interval for CoronaVac recipients is 29.1 days (SD: 4.0), ranging from 21 to 125 days.

# Figure S2. Distribution of propensity score density by the brand of vaccine before and after weighting.


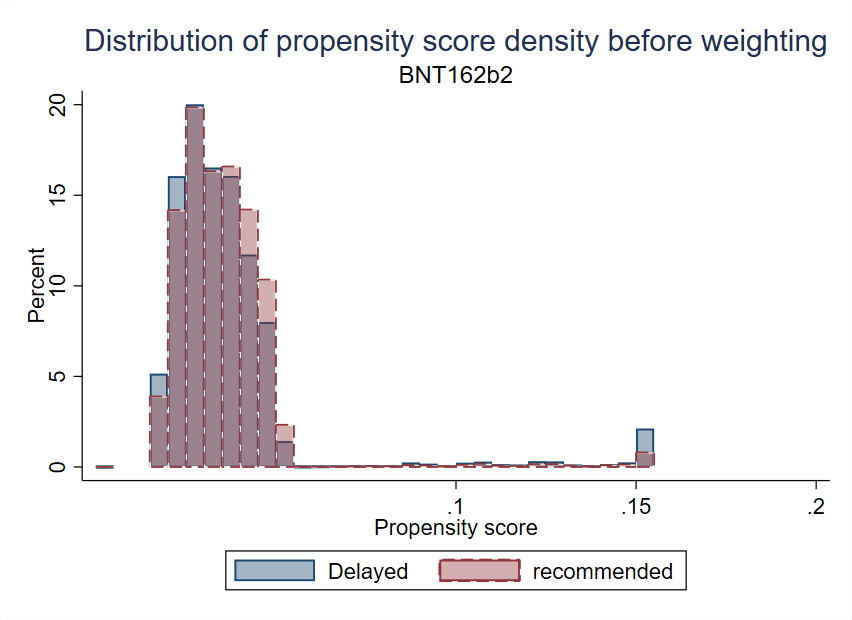

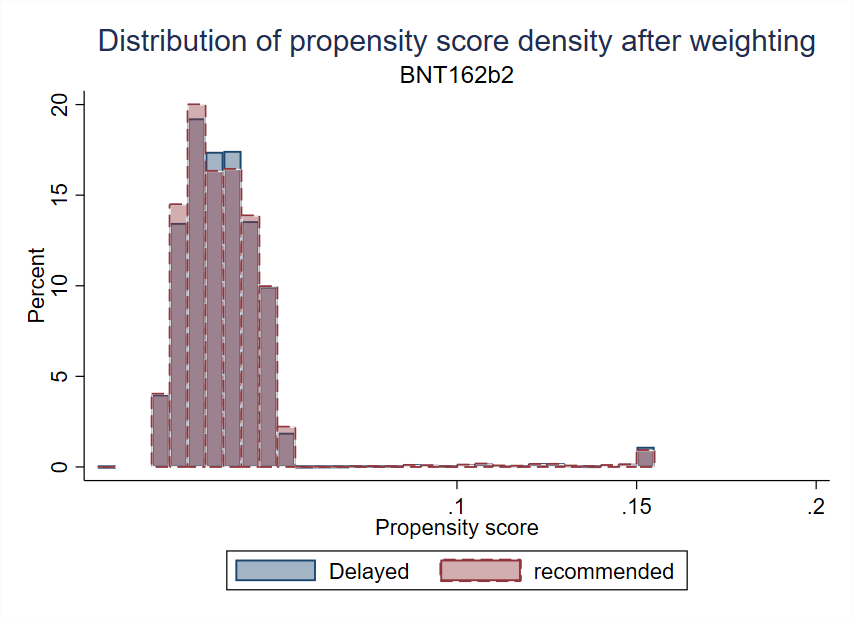


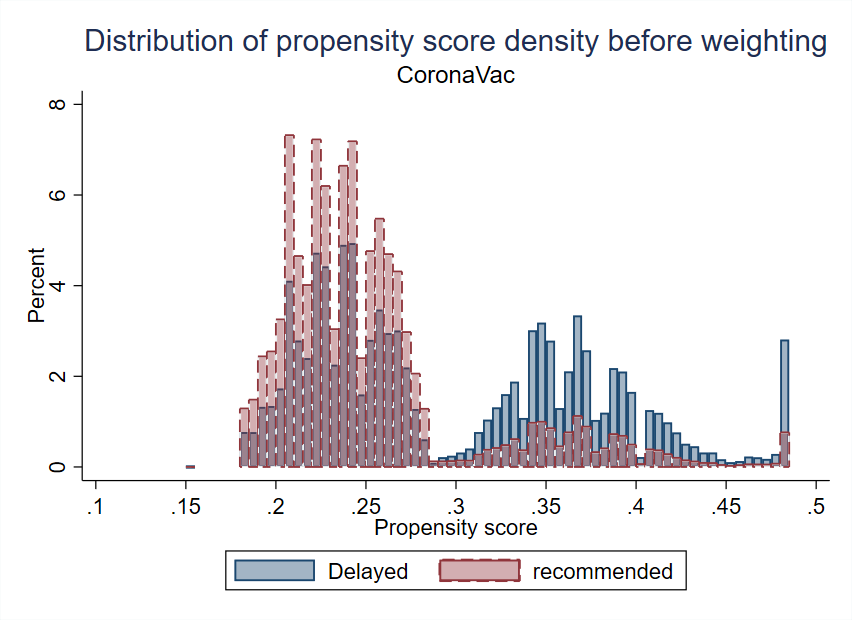

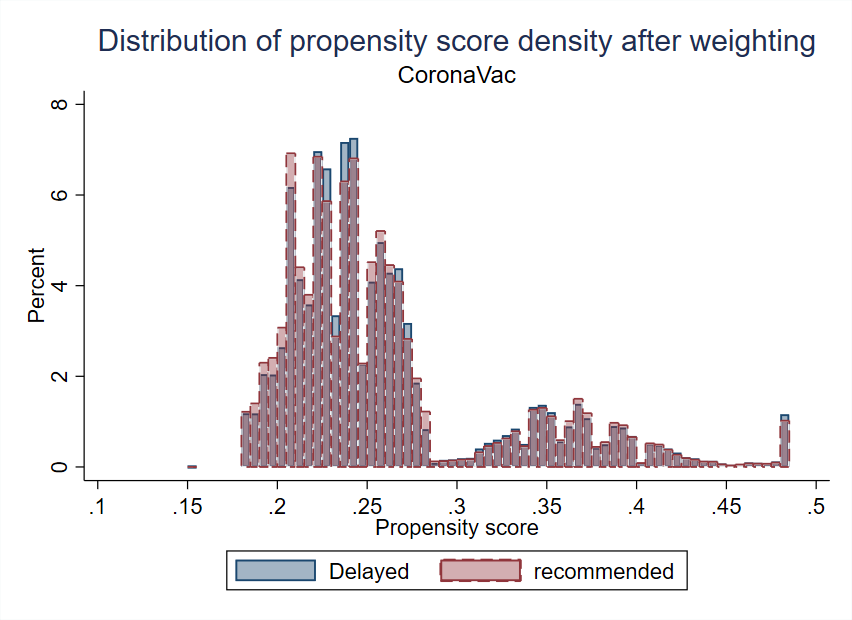

Supplement: Supplementary file 1 — Additional file 1: Impact of a delayed second dose of mRNA vaccine (BNT162b2) and inactivated SARS-CoV-2 vaccine (CoronaVac) on risks of all-cause mortality, emergency department visit, unscheduled hospitalization. Table S1. Vaccination program priority groups rollout schedule in Hong Kong. Table S2. Disease diagnosis defined by International Classification of Diseases, Ninth Revision, Clinical Modification (ICD-9-CM) diagnosis codes. Table S3. Baseline characteristics of people receiving second dose in recommended or delayed dosing interval by the brand of vaccine after the propensity score weighting. Table S4. Primary cause of unscheduled hospitalization (according to ICD-9-CM chapters) of people receiving second dose in recommended or delayed dosing interval by the type of vaccine. Table S5. Number of cases, incidence rates of all-cause mortality, emergency department visits, and unscheduled hospitalizations after second dose of BNT162b2 and CoronaVac by age. Table S6. Number of cases, incidence rates of all-cause mortality, emergency department visits, and unscheduled hospitalizations after second dose of BNT162b2 and CoronaVac in recommended, slightly delayed and delayed group. Table S7. Number of cases, incidence rates of all-cause mortality, emergency department visits, and unscheduled hospitalizations after excluding COVID-related outcomes or physical injuries. Table S8. Number of cases, incidence rates of all-cause mortality, emergency department visits, and unscheduled hospitalizations after including infinite delayers who delayed second dose for more than three months to delayed group. Table S9. Number of cases, incidence rates of all-cause mortality, emergency department visits, and unscheduled hospitalizations by using doubly robust method with inverse-probability-weighted regression-adjustment combination. Fig. S1. Distribution of second dose interval of BNT162b2 and CoronaVac recipients. Fig. S2. Distribution of propensity score density by the brand o [file 12916_2022_2321_MOESM1_ESM.docx]
